# Predicting Mental Imagery-Based BCI Performance from Personality, Cognitive Profile and Neurophysiological Patterns

**DOI:** 10.1371/journal.pone.0143962

**Published:** 2015-12-01

**Authors:** Camille Jeunet, Bernard N’Kaoua, Sriram Subramanian, Martin Hachet, Fabien Lotte

**Affiliations:** 1 Laboratoire Handicap & Système Nerveux, University of Bordeaux, Bordeaux, France; 2 Project-Team Potioc, Inria Bordeaux Sud-Ouest/LaBRI/CNRS, Talence, France; 3 Interact Lab, University of Sussex, Brighton, United Kingdom; Interdisciplinary Center (IDC) Herzliya, ISRAEL

## Abstract

Mental-Imagery based Brain-Computer Interfaces (MI-BCIs) allow their users to send commands to a computer using their brain-activity alone (typically measured by ElectroEncephaloGraphy—EEG), which is processed while they perform specific mental tasks. While very promising, MI-BCIs remain barely used outside laboratories because of the difficulty encountered by users to control them. Indeed, although some users obtain good control performances after training, a substantial proportion remains unable to reliably control an MI-BCI. This huge variability in user-performance led the community to look for predictors of MI-BCI control ability. However, these predictors were only explored for motor-imagery based BCIs, and mostly for a single training session per subject. In this study, 18 participants were instructed to learn to control an EEG-based MI-BCI by performing 3 MI-tasks, 2 of which were non-motor tasks, across 6 training sessions, on 6 different days. Relationships between the participants’ BCI control performances and their personality, cognitive profile and neurophysiological markers were explored. While no relevant relationships with neurophysiological markers were found, strong correlations between MI-BCI performances and mental-rotation scores (reflecting spatial abilities) were revealed. Also, a predictive model of MI-BCI performance based on psychometric questionnaire scores was proposed. A leave-one-subject-out cross validation process revealed the stability and reliability of this model: it enabled to predict participants’ performance with a mean error of less than 3 points. This study determined how users’ profiles impact their MI-BCI control ability and thus clears the way for designing novel MI-BCI training protocols, adapted to the profile of each user.

## Introduction

A brain computer interface (BCI) is a hardware and software communication system that enables humans to interact with their surroundings without the involvement of peripheral nerves and muscles, i.e., by using control signals generated from electroencephalographic (EEG) activity [1]. More specifically, this paper focuses on BCIs for which these control signals are sent via the execution of *mental tasks*: so-called Mental-Imagery based BCIs (MI-BCIs). MI-BCIs represent a new, non-muscular channel for relaying users’ intentions to external devices such as computers, assistive appliances, or neural prostheses (for a review, see [2]).

Since the 1990’s, many different kinds of MI-BCI have been developped [3]. Mostly, MI-BCIs have been designed for the purpose of improving living standards of severely motor-impaired patients (e.g. those with locked-in syndrome or spinal cord injuries) by enhancing their mobility autonomy and communication possibilities [1, 4, 5]. In addition to these applications, one should also note the emerging fields of BCI-based rehabilitation, e.g., for stroke rehabilitation [6, 7], and multimedia and virtual reality [8–10] for which MI-BCIs—notably those based on motor imagery—bring innovative perspectives.

Unfortunately, most of these promising technologies based on MI-BCIs cannot yet be offered on the public market since a notable portion of users, estimated at between 15 and 30%, does not seem to be able to control an MI-BCI based system [11]: this phenomenon is often called “BCI illiteracy” or “BCI deficiency”. Even for MI-BCI users who are not “illiterate”, the average performance they reach is most of the time rather low [12, 13], i.e., around 75% of classification accuracy for 2 class MI-BCIs. Nonetheless, it is important to note that around 20% of users obtain performances between 80% and 100% of classification accuracy [14] after training for two mental tasks.

It is now known that the control of an MI-BCI requires the aquisition of specific skills, and particularly the ability to generate stable and distinct brain activity patterns while performing the different Mental-Imagery (MI) tasks [1, 15]. Appropriate training is required to acquire these skills [15]. Yet, Lotte et al. [16] suggested that current strandard training protocols, which do not take into account the recommendations from psychology (such as proposing adaptive and progressive tasks or explanatory, supportive and multimodal feedback), are not appropriate, and thus might be partly responsible for BCI illiteracy and poor user performance. This hypothesis was strengthened in a recent study [17] in which a standard MI-BCI protocol [4] was tested in a BCI-free context: participants were asked to learn to do simple motor tasks (draw circles and triangles with a pen on a graphic tablet) using this standard MI-BCI training protocol. As would have been the case with MI tasks, participants had to find the right strategy (e.g., finding the right size and drawing speed) so that the motor task they were performing (i.e., drawing circles or triangles) was recognised by the system. Results showed that 15% of the participants did not manage to learn to perform these simple motor tasks (i.e. they did not find out how to adapt their strategy) using the standard training protocol, which is close to BCI-illiteracy rates. This result reinforced the idea that current standard MI-BCI protocols are not suitable for skill-learning, and emphasised the importance of working on improving them.

However, the fact that training protocols are not adapted does not explain the huge between-subject variability. Thus, this variability in MI-BCI control performance over subjects has raised questions about which parameters could help to predict users’ ability to control such a system. The training process to learn to control an MI-BCI being time- and resource-consuming, being able to predict users’ success (or failure) could avoid important loss of time and energy for both users and experimenters. From another perspective, knowing these predictors could also help understand why some people cannot learn to control an MI-BCI using standard protocols, and then guide the design of new training protocols that would be adapted to users’ relevant characteristics. So far, two kinds of predictors have been explored: neurophysiological and psychological predictors. A brief state of the art of these predictors is proposed in the following paragraphs.

Neurophysiological Predictors: Recently, evidence was presented that the amplitude of sensorimotor-rhythms (SMRs) at rest is a good predictor of subsequent BCI-performance in motor-imagery paradigms [13]. The authors proposed a new neurophysiological predictor based on the *μ* (about 9–14 Hz) rhythm over sensorimotor areas: referred to as “Blankertz’s SMR predictor” in this paper. This predictor was determined from a two minute-long recording in a “relax with eyes open” condition, using two Laplacian EEG channels. Results showed a correlation of r = 0.53 between the proposed predictor and BCI performance on a large subject data base (N = 80) which makes this neurophysiological predictor the most reliable so far. Moreover, Grosse-Wentrup et al. [18] demonstrated that the modulation of SMRs, induced by motor-imagery of either the left- or right-hand, was positively correlated with the power of frontal and occipital *γ*-oscillations, and negatively correlated with the power of centro-parietal *γ*-oscillations. Besides, Grosse-Wentrup and Schölkopf [19] showed that the power of high-frequency *γ*-oscillations originating in fronto-parietal networks predicted variations in performance on a trial-to-trial basis. As *γ*-oscillations are often associated with shifts in attention [19], the authors interpreted this finding as empirical support for an influence of attentional networks on BCI performance via the modulation of SMRs [19]. Furthermore, Ahn et al. [20] investigated the difference between BCI-literate and BCI-illiterate groups in terms of spectral band powers by comparing non-task related state (NTS) during the eyes-open state, resting but ready state (before motor imagery) and during motor imagery. They found that the BCI-illiterate group showed high *θ*- and low *α*-power levels in comparison with the BCI-literate group. Statistically significant areas were frontal and posterior-parietal regions for the *θ*-band and the whole cortex area for the *α*-band. A high positive correlation between *γ*-activity and motor-imagery performance was also shown by [21] in the prefrontal area. Finally, [22] proposed a novel predictor computed from the spectral power of pre-cue EEG data for specific rhythms over different regions of the brain. The authors argue that this predictor reflects the attentional level. Results showed that there is a significant correlation (r = 0.53) between the predictor and the cross-validation accuracies of subjects performing motor-imagery. They also found that having higher frontal *θ* and lower posterior *α* prior to performing motor-imagery, which reflects a high attentional level, may enhance the BCI classification performance. This last result seems to be in contradiction with [20]. However, the brain areas considered in these two studies are different. Ahn et al. [20] used sensori-motor areas while Bamdadian et al. [22] considered frontal theta and lower posterior alpha. Furthermore, the statistical analyses used by the authors in [20] have recently been criticised in [23]: the discrepancies in the analyses could also explain this contradiction. While the search for neurophysiological predictors seems to be a promising approach, some studies showed that the user’s psychological profile could also be an important factor influencing BCI-control performance.

Psychological Predictors: Memory span and attention were correlated to the ability to regulate slow cortical potentials (SCP) in patients with epilepsy [24]. Besides, Neumann and Birbaumer [25] found that mood together with certain predictors which were neither psychological nor neurophysiological, such as quality of caregiving, headache, sleep, and even room temperature were related to BCI performance in some patients. Nijboer et al. [26] correlated mood and motivation with SMR-BCI performance. The authors showed that higher scores of mood and mastery confidence were related to better SMR regulation abilities, whereas higher rates of fear of incompetence were correlated to lower SMR regulation abilities. Furthermore, [27] obtained a positive correlation between a Locus of control score related to dealing with technology and the accuracy of BCI control. Fear of the BCI system was also shown to affect performance [27–29]. Finally, Hammer et al. [30] showed that the psychological parameters they investigated (attention span, personality and motivation) play only a moderate role in one-session SMR-BCI control performance. However, their findings support the validity of the “Blankertz’s SMR predictor” [13] mentioned earlier (*μ* peak during relaxation) and they proposed a model for predicting SMR-BCI performance—including visuo-motor coordination (assessed with the Two-Hand Coordination Test) and the degree of concentration (assessed with the Attitudes Towards Work)—that reached significance. In a recent study, Hammer et al. [14] tested this model in a 4 session experiment (one calibration and three training sessions) within a neurofeedback based SMR-BCI context (i.e., involving no machine learning). Their results showed that these parameters explained almost 20% of the SMR-BCI performance in a linear regression. However, the first predictor, i.e., visuo-motor coordination, failed significance. With this model, the average prediction error was less than 10%.

To summarise, all the studies concerning BCI-performance predictors considered either Band Power values for SMR-BCIs, or Slow Cortical Potentials (SCP). As stated by [18], “it remains to be seen if similar results can be obtained for BCI systems not [only] based on motor paradigms”. Furthermore, most of the previous studies were based on a few runs, most of the time recorded during a one-session experiment. Yet, except for SCP-BCI [25], it has not been shown that first session performance was representative of long-term MI-BCI control performance. Indeed, first session performance can differ greatly from subsequent sessions due to several factors: (1) the fact that the classifier is trained during the first session, (2) the fact that the cap position can change, (3) the EEG-signal non-stationnarity or (4) the novelty effect. Finally, there is only one study, by Hammer et al. [30], in which psychological factors were combined with a neurophysiological predictor [13] to determine a predictive-model of motor-imagery based BCI performance.

The main contribution of this paper is to propose a predictive model of MI-BCI control performance, which was designed considering the possibility of combining several psychological and neurophysiological factors. Indeed, participants were asked to learn to perform three MI tasks, namely one motor-imagery task, i.e., left-hand movement imagination, and two non motor tasks, i.e., mental rotation and mental subtraction. Their average performance over the six sessions they attended was then set as the variable to explain in a step-wise linear regression. The scores obtained at the different psychometric tests as well as neurophysiological predictors were used as explanative factors.

## Materials and Methods

### Participants

18 BCI-naive participants (*9 females; aged 21.5 ± 1.2*) took part in this study, which was conducted in accordance with the relevant guidelines for ethical research according to the Declaration of Helsinki. This study was also approved by the legal authorities of Inria Bordeaux Sud-Ouest (the COERLE, approval number: 2015–004) as it satisfied the ethical rules and principles of the institute. All the participants signed an informed consent form at the beginning of the experiment and received a compensation of 100 euros at the end. Furthermore, in the aim of avoiding confounding factors, age [21.5 ± 1.2 year old] and educational level [14.5 ± 1.8 years of education] were controlled, which means that the ranges of these variables were low: participants were in the [20;25] year old interval and were studying at the University, for a Bachelor or Master degree. All of the participants were healthy and right handed (Harris lateralisation test [31]).

### Variables and Factors

The aim of this study was to evaluate the impact of different psychological and neurophysiological parameters on MI-BCI performance in healthy participants in order to propose a model that could predict MI-BCI performances. Thus, the effect of the scores obtained at different neuropsychological questionnaires and of the values of neurophysiological markers on the variable “MI-BCI classification performance” was evaluated.

### Experimental Paradigm

Each participant took part in 6 sessions, on 6 different days spread out over several weeks. Each session lasted around 2 hours and was organised as follows: (1) completion of psychometric questionnaires, which are described in the next section (around 30 min), (2) installation of the EEG cap (around 20 min), (3) five 7-minute runs during which participants had to learn to perform three MI-tasks (around 60 min, including breaks between the runs) and (4) uninstallation and debriefing (around 10 min). The MI-tasks (i.e., left-hand motor imagery, mental rotation and mental subtraction) were chosen according to Friedrich et al. [32], who showed that these tasks were associated with the best performance. “Left-hand motor imagery” (*L-HAND*) refers to the kinesthetic continuous imagination of a left-hand movement, chosen by the participant, without any actual movement [32]. “Mental rotation” (*ROTATION*) and “mental subtraction” (*SUBTRACTION*) correspond respectively to the mental visualisation of a 3 Dimensional shape rotating in a 3 Dimensional space [32] and to successive subtractions of a 3-digit number by a 2-digit number (ranging between 11 and 19), both being randomly generated and displayed on a screen [32].

**Fig 1:**
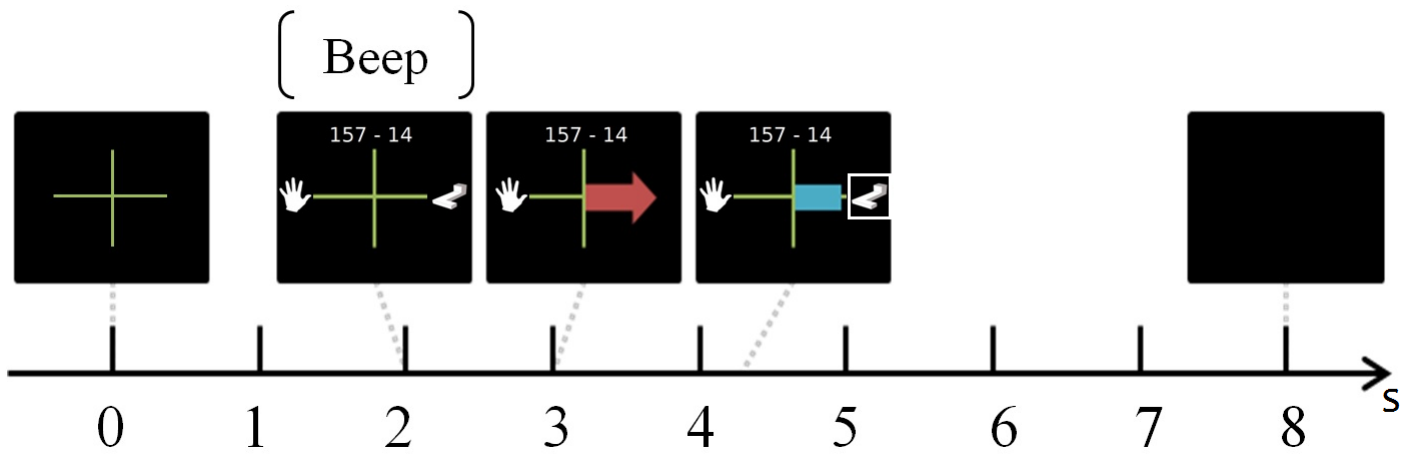
Timing of the protocol.

During each run, participants had to perform 45 trials (15 trials per task x 3 MI-tasks, presented in a random order), each trial lasting 8s (see Fig 1). At t = 0s, an arrow was displayed with a left hand pictogram on its left (*L-HAND* task), the subtraction to be performed on top (*SUBTRACTION* task) and a 3D shape on its right (*ROTATION* task). At t = 2s, a “beep” announced the coming instruction and one second later, at t = 3s, a red arrow was displayed for 1.250s. The direction of the arrow informed the participant which task to perform, e.g., an arrow pointing to the left meant the user had to perform a *L-HAND* task. In order to stress this information, the pictogram representing the task to perform was also framed with a white square until the end of the trial. Finally, at t = 4.250s, a visual feedback was provided in the shape of a blue bar, the length of which varied according to the classifier output. Only positive feedback was displayed, i.e., the feedback was provided only when there was a match between the instruction and the recognised task. The feedback lasted 4s and was updated at 16Hz, using a 1s sliding window. During the first run of the first session (i.e., the calibration run, see next Section), as the classifier was not yet trained to recognise the mental tasks being performed by the user, it could not provide a consistent feedback. In order to limit biases with the other runs, e.g., EEG changes due to different visual processing between runs, the user was provided with an equivalent sham feedback, i.e., a blue bar randomly appearing and varying in length, and not updated according to the classifier output as in [32]. A gap lasting between 1.500s and 3.500s separated each trial. At the end of the 5 runs, participants were asked to rate their arousal on the Self-Assessment Manikin scale [33] and to rate their invested mental effort on the Rating Scale Mental Effort [34].

### EEG Recordings & Signal Processing

The EEG signals were recorded from a g.USBamp amplifier (g.tec, Graz, Austria), using 30 scalp electrodes (F3, Fz, F4, FT7,FC5, FC3, FCz, FC4, FC6, FT8, C5, C3, C1, Cz, C2, C4, C6, CP3, CPz, CP4, P5, P3, P1, Pz, P2, P4, P6, PO7, PO8, 10–20 system) [32], referenced to the left ear and grounded to AFz. EEG data were sampled at 256 Hz.

In order to classify the 3 mental imagery tasks on which our BCI is based, the following EEG signal processing pipeline was used. First, EEG signals were band-pass filtered in 8–30Hz, using a Butterworth filter of order 4. Then EEG signals were spatially filtered using 3 sets of Common Spatial Pattern (CSP) filters [35]. The CSP algorithm aims at finding spatial filters whose resulting EEG band power is maximally different between two classes. Each set of CSP filters was optimised on the calibration run of each user (i.e., the first run of the first session) to discriminate EEG signals for a given class from those for the other two classes. We optimised 2 pairs of spatial filters for each class, corresponding to the 2 largest and lowest eigen values of the CSP optimisation problem for that class, thus leading to 12 CSP filters. The band power of the spatially filtered EEG signals was then computed by squaring the signals, averaging them over the last 1 second time window (with 15/16s overlap between consecutive time windows) and log-transformed. These resulted in 12 band-power features that were fed to a multi-class shrinkage Linear Discriminant Analysis (sLDA) [36], built by combining three sLDA in a one-versus-the-rest scheme. As for the CSP filters, the sLDA were optimised on the EEG signals collected during the calibration run, i.e., during the first run of the first session. The resulting classifier was then used online to differentiate between left-hand motor imagery, mental rotation and mental subtraction during the 6 user-training sessions. The sLDA classifier output (i.e., the distance of the feature vector from the LDA separating hyperplane) for the mental imagery task to be performed was used as feedback provided to the user. In particular, if the required mental task was performed correctly (i.e., correctly classified), a blue bar with a length proportional to the LDA output and extending towards the required task picture was displayed on screen and updated continuously. If the required mental task was not correctly classified, no feedback was provided, i.e., we provided positive feedback only, as in the study of Friedrich et al. [32]. To reduce between session variability, the LDA classifiers’ biases were re-calculated after the first run of the sessions 2 to 6, based on the data from this first run, as in [32]. EEG signals were recorded, processed and visually inspected with OpenViBE [37].

### Personality and Cognitive Profile Assessment using Psychometric Questionnaires

At the beginning of each of the 6 sessions, participants were asked to complete different validated psychometric questionnaires, to assess different aspects of their personality and cognitive profile, that have been related to learning in the literature. During the first session all the participants completed the same questionnaires: the information form, the *Harris Lateralisation test* and the *State Trait Anxiety Inventory*. During the other sessions (2 to 6) the administration order of the remaining questionnaires was counterbalanced for all the participants, in the aim of avoiding order effects. Thus, we ensured that the administration time of the tests did not exceed 45min per session: it lasted 30min on average. The following questionnaires were used:

- 6 subscales of the *Wechsler Adult Intelligence Scale* (WAIS-IV) [38], assessing the four IQ dimensions: similarities & vocabulary (measuring verbal comprehension abilities), digit span (measuring verbal working memory abilities), matrix reasoning (measuring perceptive reasoning abilities), coding & symbol search (measuring speed of processing abilities).
- the *Corsi Block task* [39] focuses on visuo-spatial short term and working memory abilities.
- the *Revised Visual retention test* [40] quantifies visual retention abilities as well as perceptive organisation.
- the *Learning Style Inventory* (LSI) [41] enables to identify the students’ preferred learning styles according to four dimensions: visual/verbal, active/reflective, sensitive/intuitive and sequential/global.
- the *16 Personality Factors—5* (16 PF-5) [42] measures sixteen primary factors of personality (warmth, reasoning, emotional stability, dominance, liveliness, rule-consciousness, social boldness, sensitivity, vigilance, abstractness, privateness, apprehension, openness to change, self-reliance, perfectionism and tension) as well as five global factors of personality (extraversion, anxiety/neuroticism, tough mindedness, indepedence and self control).
- the *Internal, Powerful others and Chance scale* (IPC) [43] is a multi-dimensional locus of control assessment.
- the *State Trait Anxiety Inventory* (STAI) [44] is composed of two subscales, STAI Y-A and STAI Y-B, which respectively measure anxiety as a state and anxiety as a trait. Thus, participants were asked to complete STAI Y-B at the first session only, while they were asked to complete the STAI Y-A at the beginning of each session.
- the *Bruininks-Oseretsky Test of Motor Proficiency* (BOT-2) [45] evaluates motor abilities; based on Hammer et al. [30]. We considered only some subtests evaluating bilateral and upper limb coordination as well as fine motor skills.
- the *Mental Rotation test* [46] measures spatial abilities.
- the *Arithmetic test* [38] is one of the WAIS-IV sub scales, measuring working memory abilities and more specifically the ability to concentrate while manipulating mental mathematical problems.

### Neurophysiological Predictors of BCI Performance

Different neurophysiological patterns were explored. These patterns have been proposed in the literature as being predictors of motor imagery based BCI performance. They are introduced below:

- *α*-power [8–13Hz] over each electrode, measured pre-trial (2500ms to 500ms before the instruction) and in-trial (500ms to 3500ms after the feedback start). Low *α*-power in fronto-parietal networks has been shown to be associated to a high attentional level [22, 47].
- *β*-power [16–24Hz] over each electrode, measured pre-trial and in-trial. In the paper of Ahn et al. [20], it is stated that “BCI-illiterates” have low *β*-power.
- *θ*-power [3–8Hz] over each electrode, measured pre-trial and in-trial. Low *θ*-power was related to internalised attention in [48]. High *θ*-power has also been shown to be related to cognitive, and more specifically to memory performance, when combined with high *α* power [47].
- *γ*-power over each electrode, measured pre-trial and in-trial. High pre-trial fronto-parietal *γ*-power has been associated with attentional processes [19]. Also, the ability to modulate SMR has been shown to be negatively correlated to *γ* power in occipital areas [18]. It has to be noted that muscular activity can represent a confounding factor as it is also correlated with *γ* power [18].
- the predictor proposed by Bambadian et al. [22] was calculated on pre-trial (2500ms to 500ms before the instruction). It is claimed to reflect the participant’s attentional level as the latest is, according to the literature, positively correlated to the *θ*-power and negatively correlated to both the *α* and *β*-power:
  $$\begin{array}{c} F=\frac{\sum_{c\in C_{\theta }}P_{c}^{\theta }}{\sum_{c\in C_{\alpha }}P_{c}^{\alpha }+\sum_{c\in C_{\beta }}P_{c}^{\beta }} \end{array}$$
  with *C*_*θ*_ = [*F*_3_, *F*_*z*_, *F*_4_], *C*_*α*_ = [*P*_7_, *P*_3_, *P*_*Z*_, *P*_4_, *P*_8_] and *C*_*β*_ = [*C*_*Z*_, *Cp*_*Z*_].
- the predictor proposed by Ahn et al. [20] was computed on electrodes *C*_3_ and *C*_4_ on the data of each trial (500ms to 3500ms after the feedback start):
  $$\begin{array}{c} F=\frac{w_{1}\alpha +w_{2}\beta }{w_{3}\theta +w_{4}\gamma } \end{array}$$
  with all the *w*_*i*_ = 1.
- the Blankertz’s SMR-predictor [13] certainly is the most reliable (correlation of r = 0.53 with SMR performance over a large dataset, N = 80). It is computed from a 2 min baseline in a “rest with eyes open” state using two Laplacians over the motor cortex, i.e., C3 and C4. This predictor allows to quantify the potential for desynchronisation of the SMRs at rest, which can be used as an indicator of SMR strength during the performance of motor-imagery tasks. As no 2 min baseline had been recorded with our protocol, we used all the 3 sec. pre-trial time windows of the run (3000ms before the instruction) and computed the predictor on this sequence. More precisely, we computed the power spectrum of each 2 sec time window, averaged these spectrums (i.e., over time windows), and computed the predictor on this average spectrum.

All these neurophysiological predictors except the Blankertz SMR-predictor were computed for each trial, then averaged over all trials, runs and sessions for each subject. The Blankertz SMR-predictor was computed for each run and then averaged over all runs and sessions for each subject. The relationship between these predictor values and MI-BCI performance was then investigated.

### Analyses

During each of the 6 sessions, participants performed 5 runs. However, as the classifier was updated after the first run of each session, we only used the 4 last runs (of each session) for the analyses. Thus, we considered 360 trials (15 trials x 4 runs x 6 sessions) per mental task, i.e. 1080 trials (360 x 3 MI-tasks) for each of the 18 participants. EEG data were analysed using Matlab (http://www.mathworks.com/) in order to compute the different neurophysiological patterns that could predict MI-BCI performance according to the literature. Then, these features as well as the psychometric-test results were analysed using SPSS (http://www-01.ibm.com/software/analytics/spss/) in order to find a relevant model of MI-BCI performance predictors. In particular, correlation analyses and (step-wise) linear regressions were computed as descriptive analyses. Then, leave-one-subject-out cross-validation tests were performed in order to evaluate the predictive power and the stability of the models.

## Results

### Mental-Imagery Task Performance

Eighteen participants took part in this experiment. The data of one outlier participant were rejected since, with a mean performance of 67.21%, he outperformed (by more than two SDs) the group’s mean performance over the six sessions (${\bar{X}}_{group}$ = 52.50%; *SD* = 5.62). Thus, the following analyses were based on the data of 17 subjects.

Over the six sessions, participants achieved a mean performance of $\bar{X}$ = 51.63% (*SD* = 4.39; *range*: [43.04, 60.14]). All the participants obtained performances higher than chance level, this chance level being estimated to be 37.7% of correct classification accuracy for three classes and more than 160 trials per class and *α* = 5% [49]. In the first session, mean performance was $\bar{X}$ = 51.72% (*SD* = 8.14), in the second $\bar{X}$ = 51.18% (*SD* = 6.96), in the third, $\bar{X}$ = 53.06% (*SD* = 6.04), in the fourth $\bar{X}$ = 51.57% (*SD* = 5.64), in the fifth $\bar{X}$ = 51.78% (*SD* = 6.97) and in the sixth session $\bar{X}$ = 50.49% (*SD* = 6.25). The one-way ANOVA with the *session number* as the intra-subject factor revealed no learning effect [*F*_5,96_ = 0.270, *p* = 0.928], as was generally observed for 6 sessions of training in [50]. Moreover, no gender effect [*t*_15_ = -1.733, *p* = 0.104] was noticed.

### Correlations between performance and neurophysiological predictors

**Fig 2:**
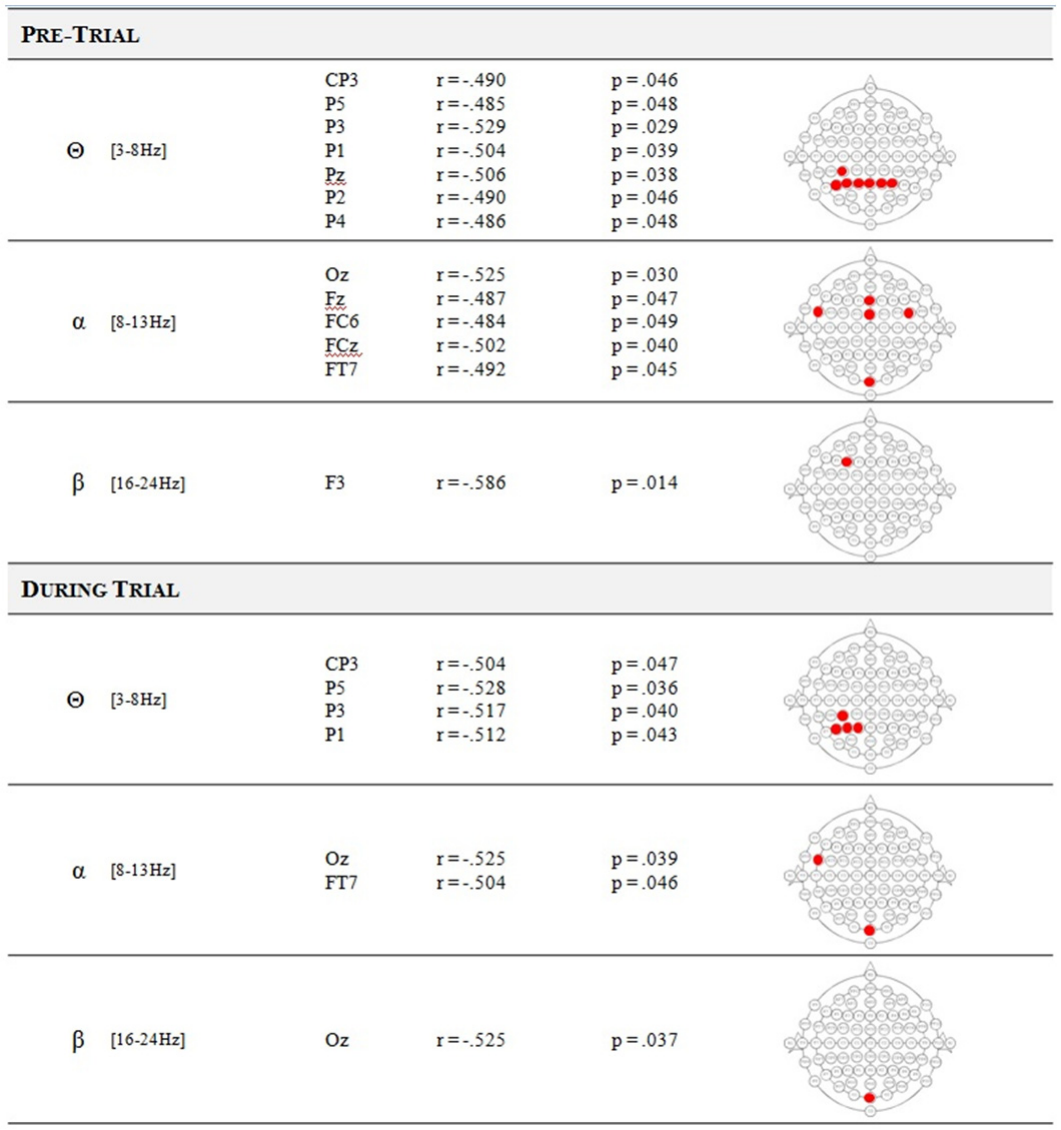
Correlations between MI-BCI performance and neurophysiological markers. Statistically significant correlations (before the correction for multiple comparisons) between MI-BCI performances and the average signal power recorded on the electrodes for the different frequency bands (*θ*, *α* and *β*) as a function of the period: pre-trial (from 2500ms to 500ms before the instruction) or during trial (from 500ms to 3500ms after the feedback start). None of these predictors reached significance after the correction for multiple comparisons.

Bivariate Pearson correlation analyses between MI-BCI performance and different neurophysiological patterns (i.e., *α*-power, *β*-power, *θ*-power, *γ*-power, Bamdadian, Ahn and Blankertz predictors) were performed. First, results showed no correlations between MI-BCI performance and the Bamdadian predictor, the Ahn predictor and the *γ*-power. Second, a tendency towards correlation was found between BCI performance and the Blankertz SMR-predictor [*r* = 0.428, *p* = 0.087]. Finally, these analyses revealed some correlations between MI-BCI performance and (1) parietal *θ*-power in both pre-trial and in-trial measurements, (2) frontal and occipital *α*-power in both pre-trial and in-trial measurements and (3) *β*-power: FT7 in pre-trial and Oz in in-trial measurements. These results are depicted in Fig 2. However, all these correlations failed to reach significance after a Positive False Discovery Rate (pFDR) correction for multiple comparisons [51].

### Correlations between performance and psychometric tests

**Fig 3:**
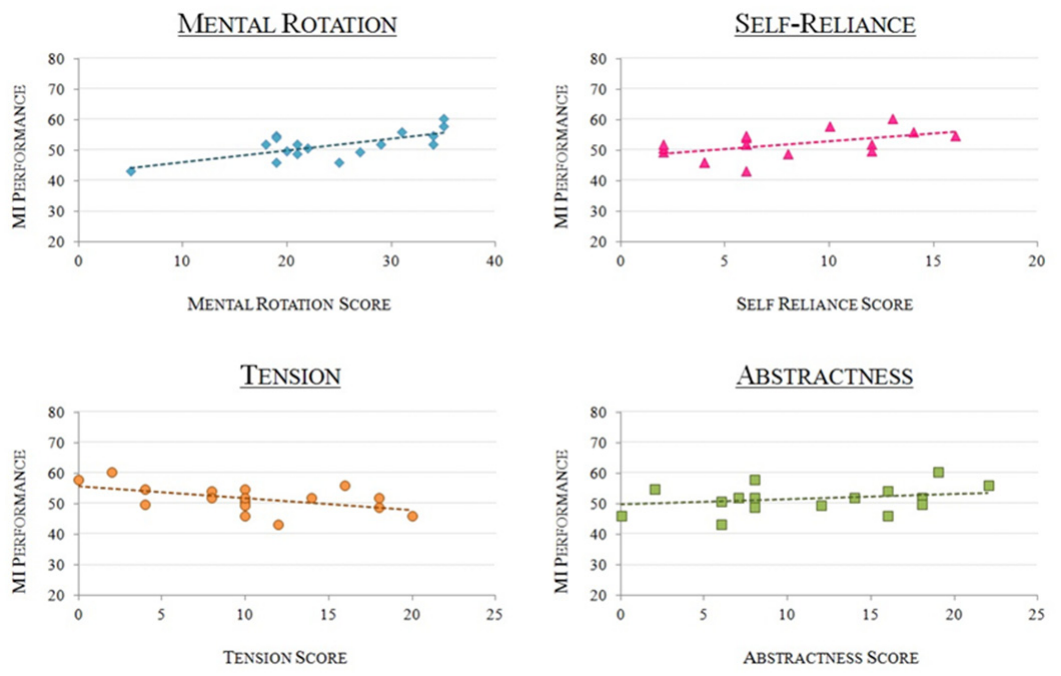
MI-BCI Performance as a function of personality profile. Graphs representing the participants’ MI-BCI performances as a function of (1) Mental Rotation scores -top left-, r = 0.696; (2) Self-Reliance -top right-, r = 0.514; (3) Tension -bottom left-, r = -0.569; (4) Abstractness -bottom right-, r = 0.526.

Bivariate Pearson correlation analyses revealed correlations between MI-BCI performance and (1) Mental Rotation scores [*r* = 0.696, *p* < 0.005], (2) Tension [*r* = -0.569, *p* < 0.05], (3) Abstractness ability [*r* = 0.526, *p* < 0.05] and (4) Self-Reliance [*r* = 0.514, *p* < 0.05] (see Fig 3). Tension, abstractness and self-reliance were assessed by the 16 PF-5. High “tension” scores reflect highly tense, impatient and frustrated personalities. The Self-Reliant trait, also called self-sufficiency, reflects the learners’ ability to learn by themselves, i.e., in an autonomous way. Finally, abstractness refers to creativity and imagination abilities. Among these four factors, only the Mental Rotation score reached significance after the Positive False Discovery Rate correction for multiple comparisons [*p* < 0.05] [51].

### First Predictive Model of MI-BCI Performance: Model ♯1

**Fig 4:**
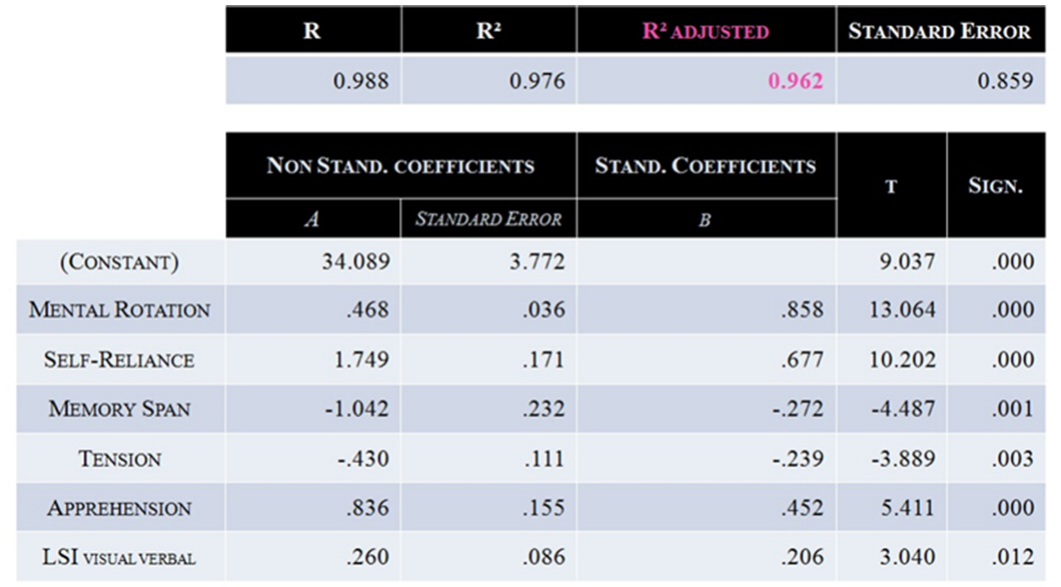
Characteristics of Model ♯1. This model included 6 factors: Mental Rotation, Self-Reliance, Memory Span, Tension, Apprehension and the “Visual/Verbal” dimension of the Learning Style. It enabled to explain 96.2% participants MI-BCI performance variance [$R_{adj}^{2}$ = 0.962, p < 0.001].

A Step-Wise Linear Regression was used in order to determine a predictive model of each user’s average MI-BCI performance obtained across the different training sessions. To reduce the dimensionality of the problem (and thus avoid the Curse-of-Dimensionality [52]), while all the psychometric test scores were used (43), only the neurophysiological predictors which were correlated with MI-BCI performance before the pFDR (20 out of ±280 neurophysiological patterns) were used as potential explanative variables in the regression. This regression resulted in a first model, called Model ♯1, including six factors [$R_{adj}^{2}$ = 0.962, p < 0.001] (see Fig 4): Mental Rotation score, Self-Reliance, Memory Span, Tension, Apprehension and the“Visual/Verbal” subscale of Learning Style. Model ♯1 explains more than 96% of the performance variance of the dataset.

In order to evaluate (1) the stability and (2) reliability of Model ♯1, step-wise linear regressions were then performed using a leave-one-subject-out cross validation process. During the *first step*, 17 models were generated, each of them based on the data of all the participants except one (i.e., the training dataset). This *first step* allowed to assess the *stability* of the model by comparing the factors included in each of the models to the ones included in Model ♯1. During the *second step*, each of these models was tested on the only participant not included in the respective training datasets (i.e., the testing dataset). This *second step* aimed at determining the *reliability* of the models. Each model generated from the training dataset enabled to determine a predicted performance as well as a confidence interval for the corresponding testing dataset. This testing dataset used the participant’s scores obtained at the psychometric tests that were included as factors in the respective training model. The model was considered reliable when the real preformance fell within the predicted confidence interval.

The first step of the the leave-one-subject-out cross validation process revealed the instability of Model ♯1. Indeed, only 5 out of 17 models included the same factors as Model ♯1. In 11 out of 17 models, 2 or more factors were different from Model ♯1. More specifically, the cross validation resulted in 13 different models for the 17 training datasets, with 27 different factors included in the different models. Among these 27 factors, 17 were present in only 1 or 2 models out of the 17.

The second step consisted in testing these 17 models on their respective testing datasets, i.e., on the only participant not included in each training dataset. Results revealed that the real performance of 9 out of 17 participants fell within the predicted confidence interval, with an absolute mean error (*Perf*_*predicted*_—*Perf*_*real*_) of 2.68 points (*SD* = 2.37, *range*: [0.38, 8.98]).

### Second Predictive Model of MI-BCI Performance: Model ♯2

**Fig 5:**
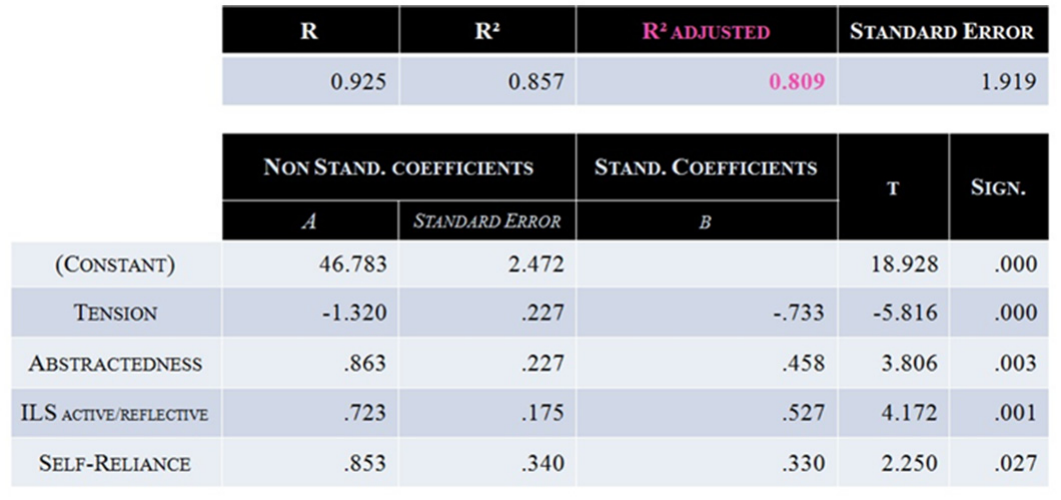
Characteristics of Model ♯2. This model included 4 factors: Tension, Abstractness, the “Visual/Verbal” dimension of the Learning Style and Self-Reliance. Abstractness, the “Visual/Verbal” dimension of the Learning Style and Self-Reliance had positive weights. Tension was the only factor to have a negative weight. This model enabled to explain 80.9% of MI-BCI performance variance [$R_{adj}^{2}$ = 0.809, p < 0.001].

In Model ♯1, the “mental rotation” factor was selected first in the regression and highly correlated with performance (r = 0.696), which demonstrates its strong influence on the model. While being consistent with the nature of the tasks performed by the participants, this strong influence was likely to hide the effect of other important factors [53, 54]. Moreover, the mental rotation score is most probably mainly related to the performance at the mental rotation imagery task, and therefore not independent of the mental tasks used in this specific BCI. Consequently, a second regression analysis was performed without the mental rotation variable. It resulted in a model, called Model ♯2 [$R_{adj}^{2}$ = 0.809, p < 0.001], described in Fig 5 and including 4 parameters: Tension, Abstractness, the Learning Style “Active/Reflective” subscale and Self-Reliance. Tension, Abstractness and Self Reliance were assessed by the 16 PF-5, whereas the “Active/Reflective” dimension is a subscale of the Learning Style Inventory.

**Fig 6:**
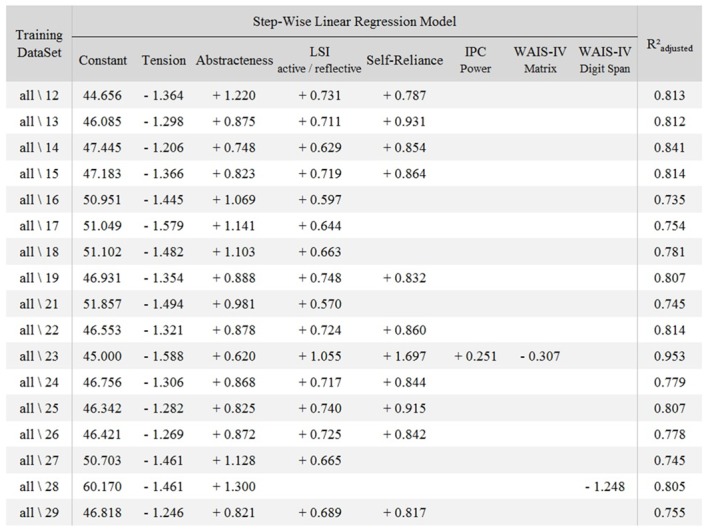
The 17 models generated from leave-one-subject-out cross validation process. The coefficients for each factor that was included in the model generated from the training datasets (*all\XX* meaning that the training dataset was composed of all the participants except XX) are detailed in each row.

As was done for Model ♯1, the stability and reliability of Model ♯2 were assessed using a leave-one-subject-out cross validation process. Results are detailed in Fig 6 which presents each training dataset, *all\XX* meaning that the training dataset was composed of all the participants except XX. The factors included in the model as a function of the dataset considered, as well as the $R_{adj}^{2}$ value of each model are also shown.

The first step allowed to evaluate the stability of Model ♯2. The same process as the one introduced in the previous section was used: 17 models were generated from the 17 training datasets, each of them including the data of all the participants except one. Results revealed that among these 17 models, 10 included exactly the same factors as the ones included in Model ♯2: Tension, Abstractness, the “Active/Reflective” Learning Style subscale and Self-Reliance. In 5 out of the 7 remaining models, only one factor, Self-Reliance, was missing. Finally, one training dataset (all∖23) induced a model including all the parameters present in Model ♯2 plus the Power dimension of the Locus of Control and the Matrix subscale of the WAIS-IV, while in another dataset (all∖28), Tension, Abstractness and the Digit Span subscale of the WAIS-IV were included.

**Fig 7:**
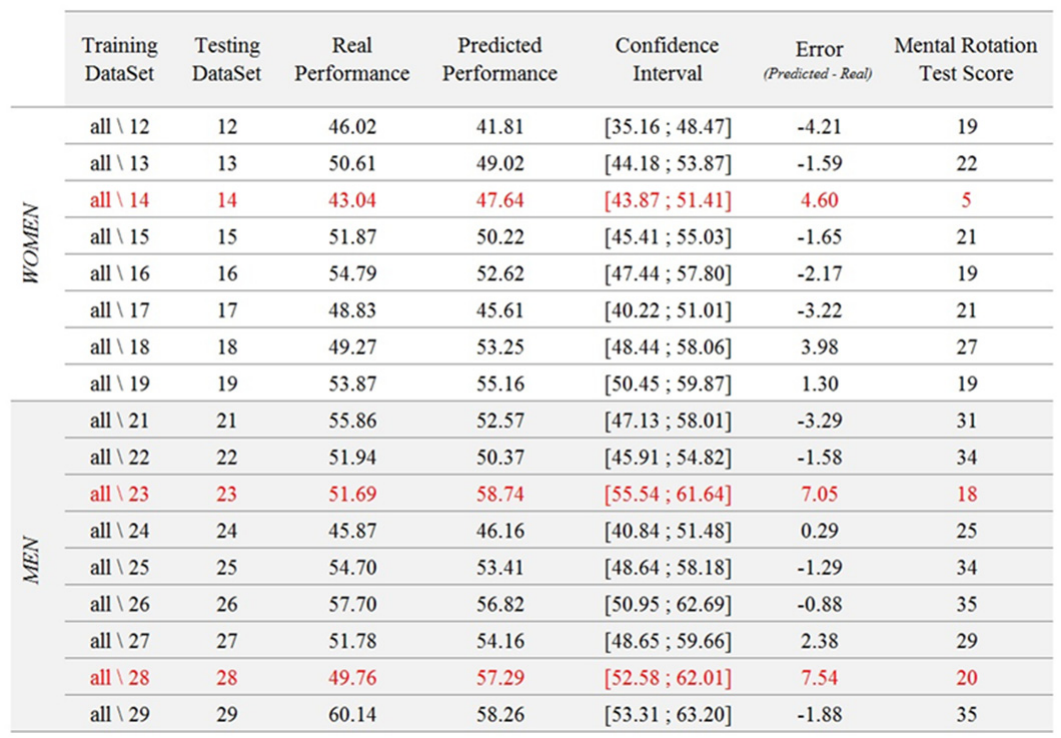
Results of the test of the 17 models generated from the training datasets on their respective testing datasets. The table shows training and testing datasets, the real performance of the testing dataset, the predicted performance of the testing dataset with the corresponding confidence interval, as well as the error of the model. Finally, in the last column the mental rotation score of the participant is outlined.

The second step allowed to determine the reliability of Model ♯2. It consisted in testing each model on the corresponding testing dataset, i.e., on the only participant whose data were not included in the training dataset. The results of this second step are detailed in Fig 7. This figure shows, for each participant (i.e., each testing dataset), (1) real mean MI-BCI performance across the 6 sessions, (2) predicted performance, with its associated confidence interval and (3) the error of the model, i.e., *Perf*_*predicted*_—*Perf*_*real*_. The average size of the confidence interval was 9.89% and the mean value of the absolute model error was 2.87%. The real performance of 14 out of 17 participants fell within the confidence interval, while the real performance of the 3 remaining participants, S14, S23 and S28, was lower than predicted.

In order to ensure that the successful prediction of BCI performance using the personality and cognitive profiles of the users was not due to chance, a permutation test was performed. The aim of this test was to estimate the true chance level in mean absolute error given our data. To do so, the first step consisted in randomly permuting the mean BCI performances of the training subjects (still using a leave-one-subject-out cross validation). The second step consisted in using the step-wise linear regression to obtain a model predicting the (random) performances of these training subjects from their (real) personality and cognitive profile, in order to simulate a random predictive model. During the third step, this model was used to predict the real BCI performance of the left-out subject. This step was repeated using each subject as the test subject, and the obtained mean absolute error over all subjects was stored. This process was repeated 1000 times, each time with a different random permutation of the subjects’ BCI performances, to estimate the performances obtained by 1000 predictive models with chance level accuracy. The obtained mean absolute errors were then sorted over the 1000 permutations in descending order, and the 99-percentile and 95-percentile were assessed to identify the chance level for *p* = 0.01 and *p* = 0.05, respectively. The results indicated that the mean absolute error of 2.87 that we obtained was better than chance with p < 0.01. This means our model can indeed generalize to new subjects and predict their MI-BCI performances from their personnality and cognitive profile. More precisely, the chance level model (obtained with the permutation test) predicted an average accuracy of 51.6331 ± 0.8620%, which corresponds to an absolute average error of 4.6859 ± 0.8752%. The chance-level predictions for each subject are displayed on Fig 7.

### Relationship between Model ♯2 and Mental Rotation Scores

**Fig 8:**
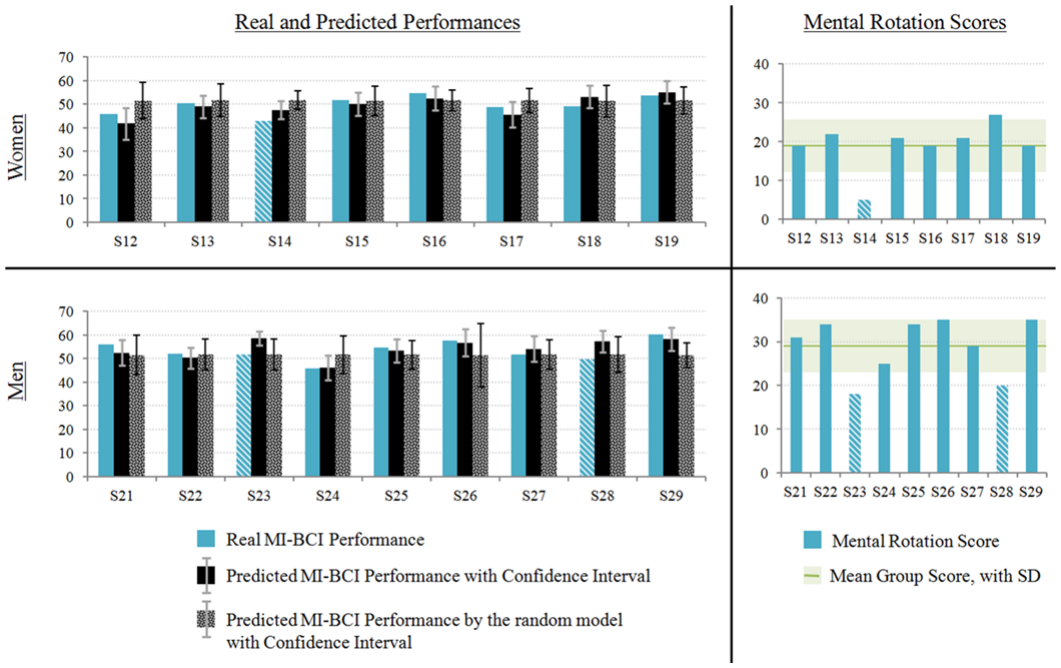
Real and predicted BCI performance as well as Mental Rotation scores according to the gender. Women’s results are shown at the top, men’s results on bottom. On the left, the graphical representation of the real (left) and predicted (right) BCI-performance of each participant, with the corresponding confidence intervals. On the right, the mental rotation scores of each participant with the horizontal line representing the mean score of the group. The three participants for whom the model overrated the performance are those with the lowest mental rotation scores (striped participants).

Fig 8 outlines women’s results on top and men’s results on the bottom at both the MI-tasks (left) and mental rotation test (right). First, graphs on the left represent each participant’s real (left) and predicted (right) performance for each participant, with the corresponding confidence intervals. These graphs show that the real performance value of 14 out of 17 participants fell within the predicted confidence interval, while it was lower for only 3 participants: S14, S23 and S28. Second, graphs on the right represent the Mental Rotation scores for all the participants. Women and men were separated due to the important gender effect associated with this test [46]. Women’s mean score is 19.13/40 (*SD*: 6.29, *range*: [5, 27]). Men’s mean score is 29/40 (*SD*:6.56, *range*: [18, 35]). Women’s and men’s mean scores are represented as a horizontal line on the graphs on the right of Fig 8. The rectangle surrounding this line represents the mean ± 1SD interval. Only 3 participants, one woman and two men, are below this interval: S14, S23 and S28.

It is noticeable that the same participants, i.e. S14, S23 and S28, (1) had lower real MI-BCI performance than the one predicted by the model and (2) had lower mental rotation scores than the average.

## Discussion

In this paper, we proposed a predictive model of MI-BCI performance based on the data of 17 participants. The important number of runs (30, spread over 6 sessions) attenuated the between-session variability (which could be due, e.g., to fatigue or motivation fluctuation, cap position variation, etc.) and thus enabled to more precisely estimate the participants’ actual long-term ability to control an MI-BCI. For the first time, performance predictors were not determined in a context of pure motor-imagery, since participants were asked to perform one motor imagery task -left-hand movement imagination- as well as two non-motor MI-tasks -mental rotation and mental subtraction-.

Five major results were obtained. The first is the strong correlation between MI-BCI performance and mental rotation scores. The second major result is the fact that, despite an apparent consistent relation between MI-BCI performance and frontal *α* and parietal *θ*-power which could suggest a role of attention processes, no significant correlation was revealed after the correction for multiple comparisons and these predictors were not selected in the regression. Thus, the considered predictors seem not to be robust nor relevant enough to predict MI-BCI performance over multiple sessions. Two plausible explanations of this result are the fact we considered 6 sessions whereas these neurophysiological predictors were computed, on the literature, based on one single session, and also the fact our paradigm involves three different MI-tasks, whereas only motor-imagery was considered in the studies from which the neurophysiological predictors were extracted. What is more, since participants were asked to perform one motor imagery task, it is interesting to notice the tendency towards a correlation between the Blankertz’s SMR-predictor and MI-BCI performances which strenghtens the reliability of this predictor for SMR modulation abilities. The fact this predictor is not significantly correlated with MI-BCI performance could also be partly due to our experimental protocol. Indeed, as no 2 minute-long baseline was recorded the predictor was computed based on the concatenation of all the 3 second-long pre-trials of the runs, which could impact its performance. The third result is the definition of Model ♯1 which explained more than 96% of the variance of participants’ MI-BCI performance. This model was composed of six factors: mental rotation, self-reliance, visuo-spatial memory span, tension, apprehension and the “visual/verbal” dimension of the learning style. The main flaw of Model ♯1 was its instability, revealed by the cross validation process. This instability could be due to the important role of the mental rotation factor in the MI-BCI performance prediction. Indeed, its strong correlation with MI-BCI performance could prevent other important factors from being expressed in the regression. Thus, the fourth major result is Model ♯2, from which the mental rotation factor was excluded. Model ♯2 explained more than 80% of MI-BCI performance variance and was composed of four factors: tension, abstractness, self-reliance and the “active/reflective” dimension of the learning style. This model appeared to be both stable and reliable to predict MI-BCI performance. It should be noted that since we averaged the BCI performances over the 6 sessions, the performance variance across subjects was rather low. As such, although our model predicted the performance significantly better than chance, the obtained error rate was not that low as compared to that obtained by a random model. Nonetheless it was still better, and, more importantly, it enabled us to identify the relevant factors (cognitive profile and personality) linked to BCI performances. Finally, the fifth and very interesting result is the complementarity between Model ♯2 and mental rotation scores. Indeed, the only participants for whom Model ♯2 failed, by overrating their performances, were the participants with a very low mental rotation score. These results are discussed in the following paragraphs.

A first very interesting result is the prominent role of mental rotation scores: this factor is highly correlated with MI-BCI performance, is the first one to be selected in Model ♯1 and brings relevant additional information to Model ♯2 to predict MI-BCI performance. Mental rotation scores reflect spatial abilities [55], i.e., the capacity to understand and remember spatial relations between objects. Mental rotation, and thus spatial abilities, are intimately related with the three mental imagery tasks considered in this study. First, it is obviously related with the mental rotation task. Second, [56] showed that children confronted with difficulties to perform arithmetics also had low spatial abilities. Third, the mental rotation test is actually used to evaluate motor imagery abilities in healthy subjects and patients with brain injuries [57]. The close relationship between mental rotation and the three MI tasks could explain the strong implication of spatial abilities in participants’ capacity to perform the MI tasks proposed to control a BCI system. This relationship suggests that it would be interesting to consider each MI task independently. However, given the protocol and the kind of classifier used, doing so would most probably provide biased results and/or results that make little sense. Indeed, 3 “‘one vs all”’ linear discriminant analysis (LDA) classifiers were used, which means that each classifier was trained to discriminate the targeted MI task from the other two. Thus, the feedback (blue bar) was not informing the user about how well he was performing the target MI task, but how much this target MI task was distinguishable from the other two. Thus, analysing the performances “‘one MI task vs. one MI task”’ would make little sense, as this was not what the user was trained to do. We could have trained offline new classifiers to discriminate “‘one MI task vs. rest”’ to know how well the different MI tasks were performed independently from the others. But the performances could be very different from the ones presented to the user. For instance, an MI-task could be associated with good performances when using a “‘one vs. all”’ classifier (because it is well distinguishable from the other MI tasks) and at the same time associated with bad performances when using a “‘one vs. rest”’ classifier (because the brain activity associated with this MI task is close to the resting state). In such a case, the participant would not have put much effort in trying to improve his performance while doing this MI task because he thought he was managing well and so it does not make sense to study his performance in another context (i.e., with another classifier) as the participant did not receive any feedback enabling him to know that he had to adapt his strategy.

Two other personality factors were strongly correlated with MI-BCI performance and are included in both models: *tension* and *self-reliance*. The *tension* dimension reflects highly tense, impatient and frustrated personalities while the *self-reliance* dimension, also called self-sufficiency, reflects the learner’s ability to learn by themselves, i.e., in an autonomous way. Both were assessed using the 16 PF-5 questionnaire. MI-BCI performance appeared to be negatively correlated with the *tension* dimension and positively correlated with the *self-reliance* dimension. These factors have been shown to be related to the nature of MI-BCI training which is a *distant learning*, i.e., a learning occuring in a context free of social interaction (the learner interacts with a computer, there are no teachers or students). Indeed, on the one hand, [58] showed that learners easily feel confusion, frustration and anxiety when confronted to distant education due to the lack of feedback from an instructor, compared to classic classroom education situations. Therefore, it seems relevant that learners with highly tense personalities encounter difficulties in learning tasks based on distance education such as the one presented in this study. On the other hand, in [59], autonomy is presented as being of utmost importance in independant learning, and thus in distance learning. During MI-BCI training, users have to lead important metacognitive processing to identify knowledge and strategies allowing them to optimise their performances. As a consequence, users with low *Self-Reliance* scores may have difficulty when confronted with MI-BCI training protocols, because they need more guidance about strategies and key steps to carry out during a training session. To summarise, it seems users with high “Tension” and low “Self-Reliance” traits may need a social presence and emotional feedback to improve their control performance. An alternative hypothesis could also be that users that are more self-reliant may comply better with the BCI tasks, i.e., they really and conscientiously perform the required tasks—which other users might not do as well—which in turns leads to higher classification accuracy. However, since we do not have the ground truth of whether users did comply with the required tasks, we cannot verify this hypothesis. This seems nonetheless a less likely hypothesis than the ones related to distant learning, which are more theoretically solid.

The *abstractness* dimension of the 16 PF-5 was also correlated with MI-BCI performance and included in Model ♯2. Abstractness refers to creativity and imagination abilities. It has been reported that creative people frequently use mental imagery for scientific and artistic productions [60] which could explain why participants with high abstractness abilities are more used to performing mental imagery.

The other factors included in Model ♯1 and Model ♯2 were not (linearly) correlated with MI-BCI performance. First, in Model ♯1, three additional factors were included: memory span (assessed by the Corsi block task), which had a negative impact on performance, apprehension (dimension of the 16 PF-5) and the “Visual/Verbal” subscale of the Learning Style Inventory, both of them having had a positive impact on participants’ MI-BCI performance. The instability of Model ♯1 made the inclusion of these factors anecdotal. However, concerning Model ♯2, the last factor, i.e., the “Active/Reflective” dimension of the Learning Style Inventory does not seem to be anecdotal as it was also included in 16 out of the 17 models generated during the cross validation process. This “Active/Reflective” dimension seems to be an important factor even if it is not linearly correlated to MI-BCI performance. Thus, active learners appear to be more efficient in learning to control an MI-BCI. The “Active/Reflective” dimension considers the complex mental process that allows converting perceived information into knowledge. This process can be of two categories: active experimentation or reflective observation [61]. While active learners like testing and discussing the information, reflective learners need more time to think and examine it introspectively. As stated in [61], reflective learners need the opportunity and time to think about the information being presented to achieve a good level of performance. Yet, in current standard protocols like the one used in the present study, participants only have four seconds to perform each MI-task proposed. Another characteristic of active learners is the fact they are more effective when they “learn by doing”. Yet, [62] showed that motor-imagery performances are higher when the subjects use active kinesthetic movement imagination strategies. It could also explain the positive impact of the “Active” trait on MI-BCI performance.

The final result is of utmost interest and concerns the complementarity of Model ♯2 with the mental rotation score. Indeed, results show that 14 out of 17 participants achieved a real MI-BCI performance that fell within the predicted confidence interval generated from the step-wise linear regression using a cross-validation process. For the 3 other participants, the real performance was below this confidence interval. Yet, these three participants were also the ones with the lowest mental rotation scores. This means that the only times the model failed by overrating a participant’s performance, was when this participant’s spatial abilities were significantly lower than average. This result suggests that the factors included in Model ♯2, i.e., tension, abstractness abilities, the “active/reflective” dimension and self-reliance are highly reliable to predict MI-BCI performance while the user has *normal* to *good* spatial abilities. However, if the user’s spatial abilities are too low, this factor’s weight being the most influencial, it has the upper hand and decreases MI-BCI performance. In this case, the model’s overrating of MI-BCI performance can be anticipated. Considering both Model ♯2 and spatial abilities together has the advantage of taking into account all the parameters that seem to impact MI-BCI performance.

This model should now be tested on larger and more heterogenous populations (for instance to have a wider range of performance) in order to confirm (or refute) its validity, and adjust the value of the coefficients associated with each factor. Nonetheless, this model offers promising perspectives for improving MI-BCI training protocols.

This study has highlighted the huge impact of spatial abilities on MI-BCI performance. Future work will consist in designing new kinds of MI-BCI training protocols aiming at improving users’ spatial abilities, prior to MI-BCI use. Concretely, based on his/her basic spatial abilities, the user will be provided with specific exercices. The difficulty of these exercices will increase gradually, according to the user’s results, to end with complex MI tasks allowing to control an MI-BCI. It would also be interesting to adapt the MI-tasks to each user so that they are optimal for each of them.

Furthermore, in order to take into account the personality factors related to MI-BCI performance, a virtual learning companion will be developped. It will be able to provide the user with (1) cognitive support (e.g., by proposing examples) in the case of students with low abstractness abilities, (2) emotional and social support, notably social presence by giving advice and collaborating during the training procedure, for users with high “tension” and low “self-reliance” scores.

We hypothesise that by combining tindividualised training to improve the users’ spatial abilities with a virtual learning companion providing a user-specific support in an intelligent tutoring system [63], MI-BCI training will be more user friendly.

This improved training protocol could potentially increase acceptability and accessibility of MI-BCI based technologies, which are extremely promising for improving living standards of severly motor disabled patients and their families, for stroke rehabilitation, for leisure (e.g., video games) or for education.
