# Correction: Predicting Mental Imagery-Based BCI Performance from Personality, Cognitive Profile and Neurophysiological Patterns

**DOI:** 10.1371/journal.pone.0282281

**Published:** 2023-02-23

**Authors:** Camille Jeunet, Bernard N’Kaoua, Sriram Subramanian, Martin Hachet, Fabien Lotte

Data reported in this article [1] for 2 of the 18 subjects are incorrect due to a computational error. The abstractedness scores (STEN) are reported as 8 where the actual scores were 4. Due to this error, the Abstractedness factor was included in the predictive model of MI-BCI performance when it should not have been. Other factors included in the model are not affected by this.

With this error corrected, a stable model is obtained, including the following variables: Mental Rotation—Self-Reliance—Apprehension—Visual/Verbal of the learning style. The revised model has equivalent (or slightly better):

- prediction performances—*R²*_*adj*_-model#1-original = 0.962; *R²*_*adj*_-model#2-original = 0.809; *R²*_*adj*_-model-revised = 0.878
- stability—number of cross-validation models including the same factors as the global model: model#1-original: 5/17, model#2-original: 10/17, model-revised: 13/17
- reliability—number of tests for which the real performance of the subject is included in the predicted confidence interval: model#1-original: 9/17, model#2-original: 14/17, model-revised: 12/17

These results are depicted in updated Figs 4, 6, 7, and 8 included with this notice and further described in the following paragraphs. The statistical significance of the model remains unchanged (<0.001). The underlying data supporting the published and revised results are provided in S1 File.

The model explains more than 87% of the variance of the performance of the dataset and was obtained without including memory span scores (Corsi test [2]), as those scores appeared to impair the model’s predictive performance, stability, and reliability.

The updated model, while different from the previous one (Model #1 in [1]), remains consistent with that original model:

- inclusion of the “mental rotation” and “self-reliance” factors.
- inclusion of the “apprehension” factor instead of the “tension” factor, but both are part of the same global dimension of the 16PF-5, namely “anxiety”; also, “tension” remains correlated with MI-BCI performance.
- removal of the “abstractedness” factor (which is the factor for which there was a computation mistake) from the model.
- inclusion of a different dimension of the same test, the learning style inventory: “visual/verbal” instead of “active/reflective”. This is the main difference between both models. Nonetheless, this difference induces no modifications of the main message of the paper.

The stepwise linear regressions were repeated using a leave-one-subject-out cross validation process, starting with 17 models in the first step. This reanalysis replaces the analysis that was reported in the “First Predictive Model of MI-BCI Performance: Model #1” section of the published article [1]. Among the 17 models, 13 included exactly the same factors as the ones included in the “global” model: Mental Rotation, Self-Reliance, Apprehension and the Visual-Verbal dimension of the Learning Style. While the other 4 models also included the Mental Rotation and Self-Reliance factors, they revealed more variability. Of these four models that revealed more variability, one included only these two factors (Mental Rotation and Self-Reliance). A second one included the “Apprehension”, “Tension”, and “Openness to change” factors, while a third one included the “Apprehension”, “Openness to change”, “Reasoning”, and “Independence” factors (in addition to the “Mental Rotation” and “Self-Reliance” factors). Finally, the fourth one included the four factors of the general model to which the “Reasoning”, “Independence”, “Tension”, “Benton”, “Sensitivity”, “Extraversion”, “Liveliness” and “Sensing/Intuitive” dimension of the learning style were added. In these 3 last cases, an over-fitting seemed to occur, with models explaining between 94% and 100% of the variance of the dataset.

The second step consisted in testing these 17 models on their respective testing datasets, i.e., on the only participant not included in each training dataset. Results revealed that the real performance of 12 out of 17 participants fell within the predicted confidence interval, with an absolute mean error (Perf_predicted_—Perf_real_) of 1.00 point(s) (SD = 0.62, range: [0.06, 2.21]). Regarding the 5 other participants, the absolute mean error was of 4.91 points (SD = 1.98, range: [2.75, 8.08]). The real performance of two of them was less than 0.2 points higher than the upper bound of the predicted confidence interval (95%). Regarding the three other participants, who are those for whom the model over-fitted, the confidence intervals were very narrow (3.12 points on average, while the average size of the other intervals was 8.34 points). This over-fitting, combined with the resulting narrowness of the confidence interval, resulted in erroneous predictions.

In the original article [1], two models were introduced. As the first model (including the “mental rotation” factor) was neither stable nor reliable, a second model was proposed (not including the “mental rotation” factor). With the STEN errors corrected, the new initial model (that includes the “mental rotation” factor) is stable and reliable, and so it is no longer relevant to introduce a second model or to analyze the relationship between “mental rotation” factor and model 2. The article sections titled “Second Predictive model of MI-BCI Performance: Model #2” and “Relationship between Model #2 and Mental Rotation Scores,” and Fig 5 which details the characteristics of Model #2, should therefore be disregarded.

The main message of the paper, namely the fact that spatial abilities, self-reliance, and anxiety have a major impact on MI-BCI performance and learning, is not affected by this mistake.

**Fig 3:**
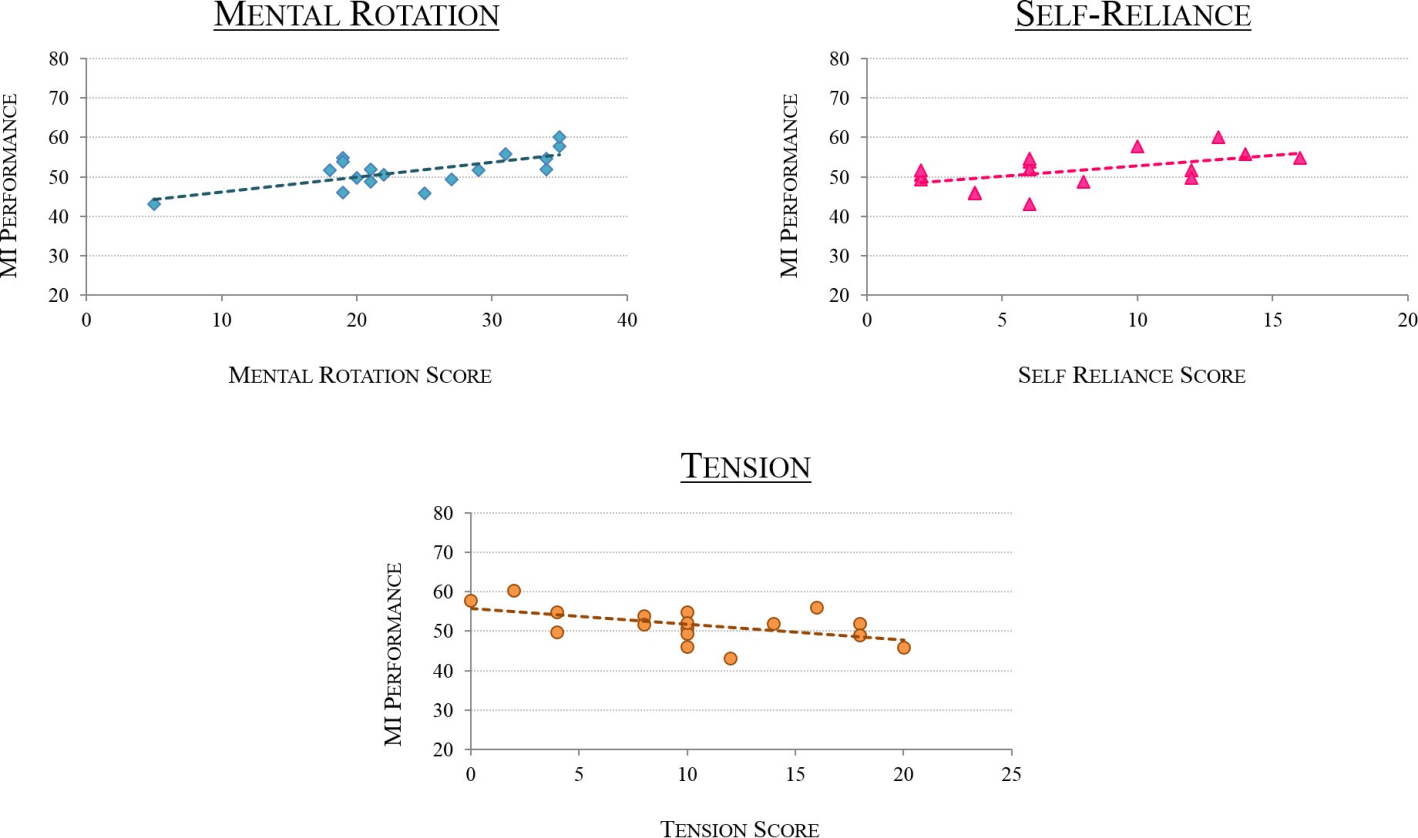
MI-BCI Performance as a function of personality profile. Graphs representing the participants’ MI-BCI performances as a function of (1) Mental Rotation scores -top left-, r = 0.696; (2) Self-Reliance -top right-, r = 0.514; (3) Tension -bottom-, r = -0.569.

**Fig 4:**
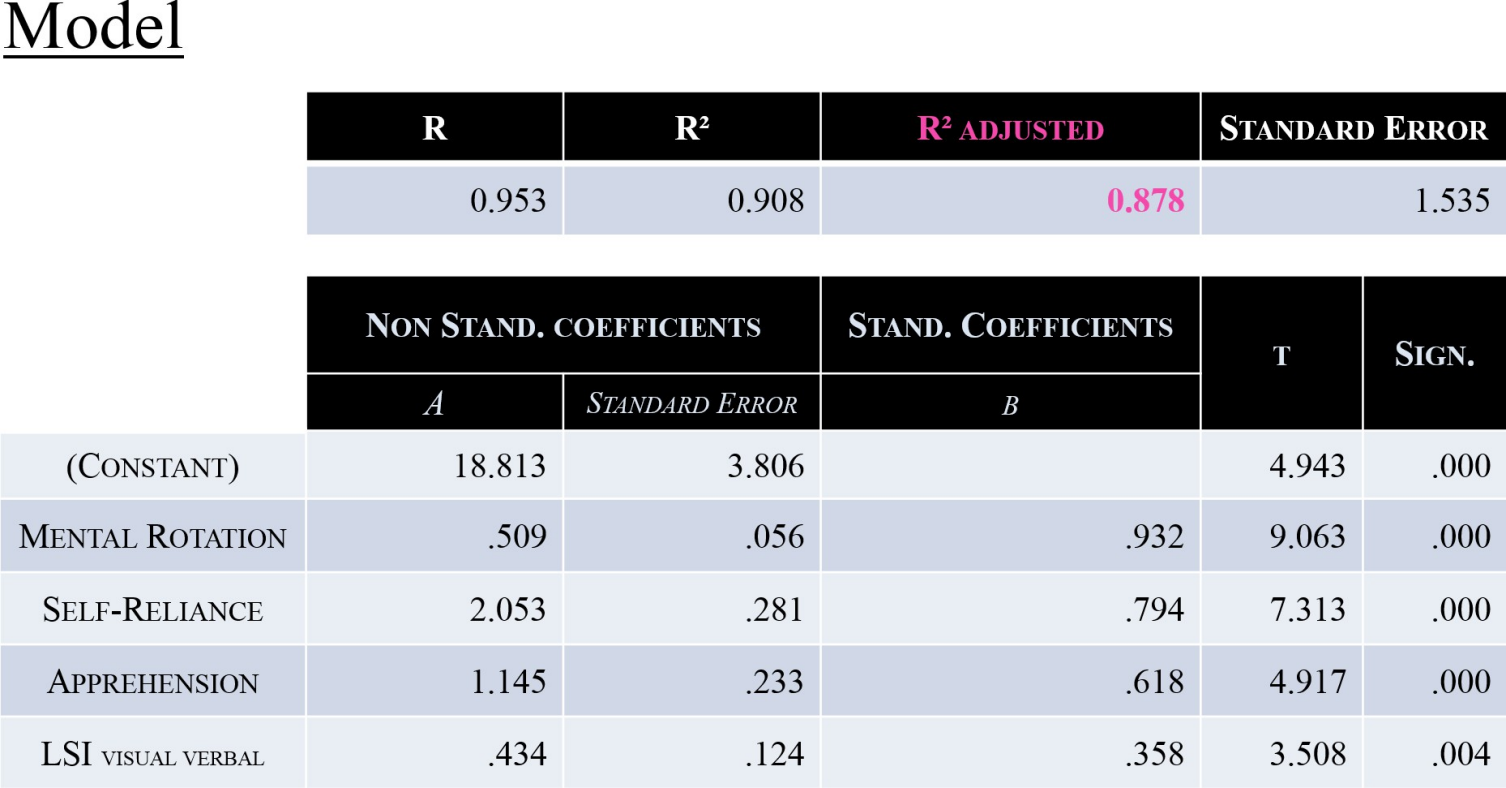
Characteristics of the Model. This model included 4 factors: Mental Rotation, Self-Reliance, Apprehension and the “Visual/Verbal” dimension of the Learning Style. It enabled to explain 87.8% of participants’ MI-BCI performance variance [*R*^*2*^_*adj*_ = 0.878, p*<*0.001].

**Fig 6:**
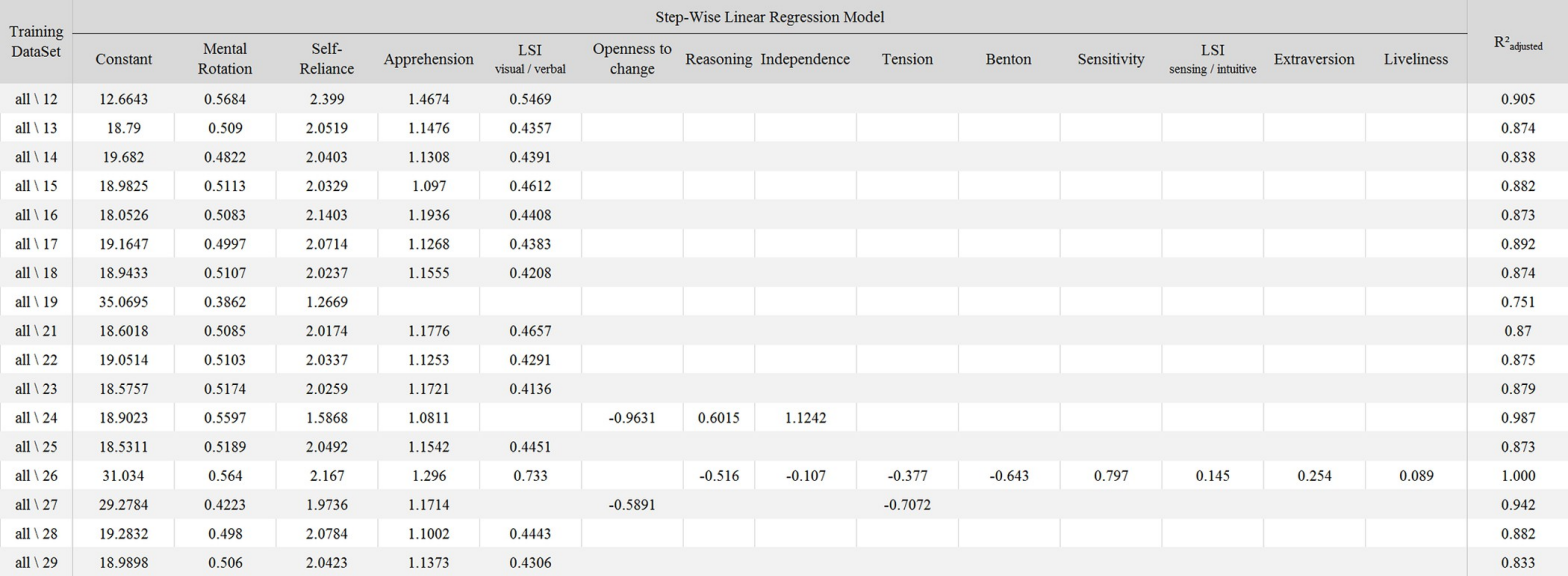
The 17 models generated from leave-one-subject-out cross validation process. The coefficients for each factor that was included in the model generated from the training datasets (*all\XX* meaning that the training dataset was composed of all the participants except XX) are detailed in each row.

**Fig 7:**
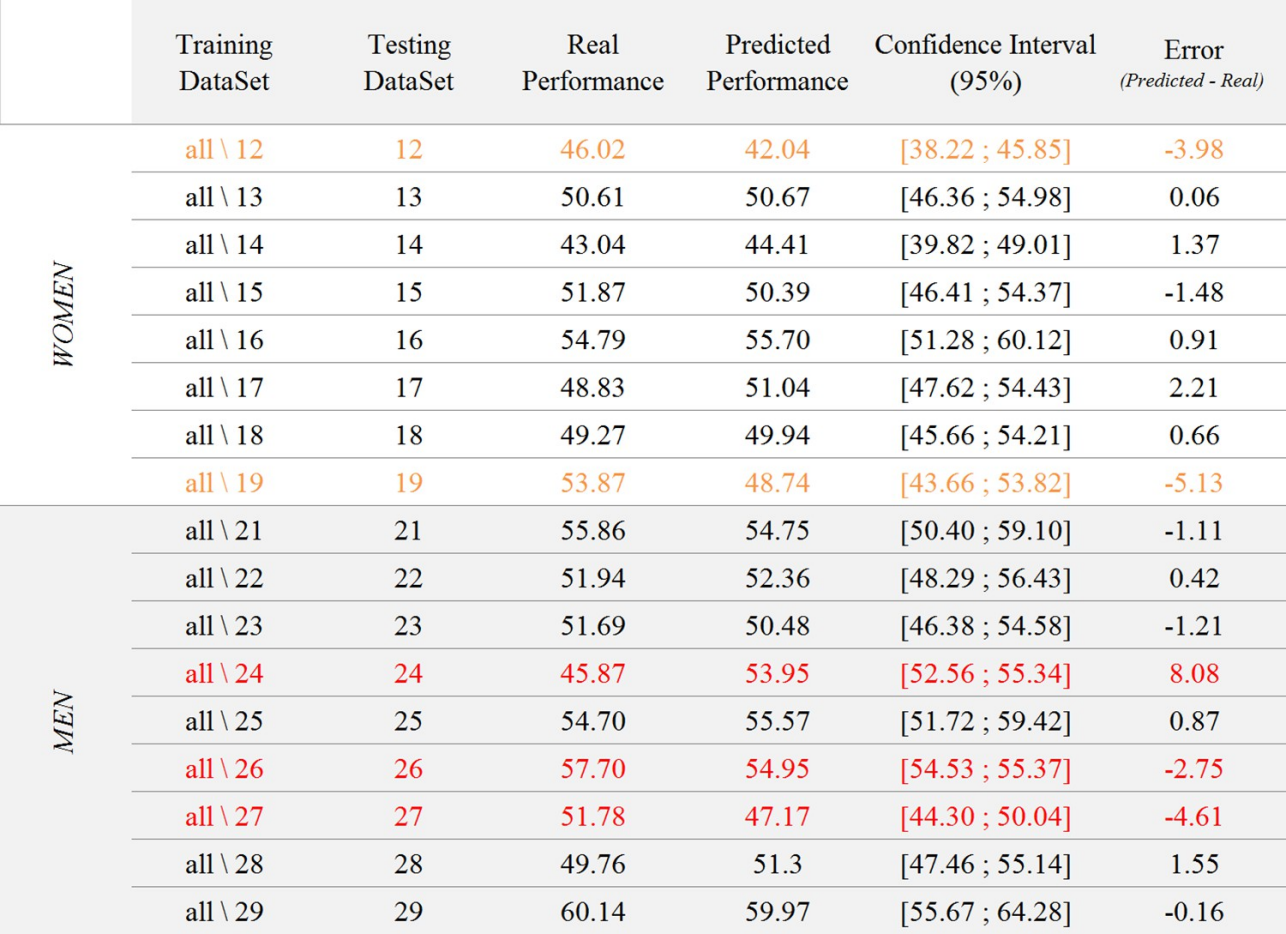
Results of the test of the 17 models generated from the training datasets on their respective testing datasets. The table shows training and testing datasets, the real performance of the testing dataset, the predicted performance of the testing dataset with the corresponding confidence interval, as well as the error of the model.

**Fig 8:**
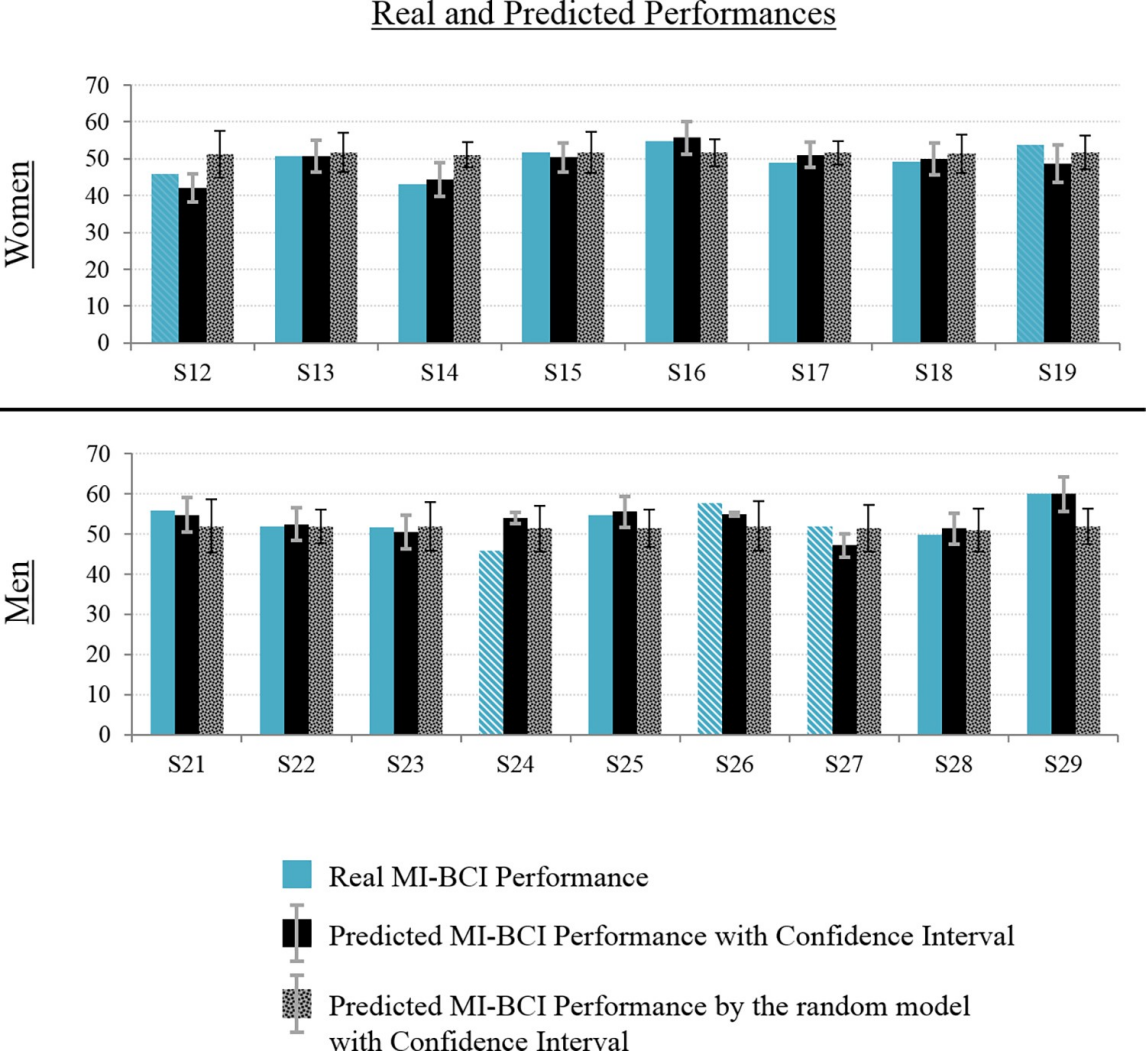
Real and predicted BCI performance. Women’s results are shown at the top, men’s results on bottom. We propose a graphical representation of the real (left), predicted (middle) and “chance-level predicted” (right) BCI-performance of each participant, with the corresponding confidence intervals.

## Supporting information

S1 File Raw data. Table containing all the experimental data, namely each participant’s BCI performances, psychometric scores, and neurophysiological predictors scores.
(XLSX)

## References

[1] Jeunet C, N’Kaoua B, Subramanian S, Hachet M, Lotte F (2015) Predicting Mental Imagery-Based BCI Performance from Personality, Cognitive Profile and Neurophysiological Patterns. PLoS ONE 10(12): e0143962. doi: 10.1371/journal.pone.0143962 26625261 PMC4666487

[2] Berch DB, Krikorian R, Huha EM. The Corsi block-tapping task: Methodological and theoretical considerations. Brain and cognition. 1998;38:317–38. doi: 10.1006/brcg.1998.1039 9841789

